# 

**Published:** 1878-05

**Authors:** 


### CONTENTS:

ORIGINAL LECTURES:
Paralysis and Convulsions as Effects of Diseases of the Base of the Brain. By Dr. Brown-Sequard .......... 449
ORIGINAL COMMUNICATIONS:
A Clinical Study of Molluscum Contagiosum. By G. H. Fox, M. D. .......... 460
Does Acute General Dropsy ever occur as an Idiopathic Disease. By J. A. Goldsberry, M. D. .......... 475
Measurements of Extremities. By John Bartlett, M. D. .......... 484
Danger from Hypodermic Injection of Morphia. By E. Fletcher Ingals, M. D. 491
How to Save the Perineum. By Edw. Warren Sawyer, M. D. .......... 496
Report of Seven Cases of Tracheotomy. By E. W. Lee, M. D. .......... 501
The History of a Case of Gunshot Wound of the Bladder. By Franklin Staples, M. D. .......... 510
CLINICAL REPORTS:
United States Marine Hospital, San Francisco .......... 514
Notes from Private Practice .......... 516
SOCIETY REPORTS:
Chicago Medical Society .......... 522
Society of Physicians and Surgeons. .......... 526
REVIEWS AND BOOK NOTICES .......... 528
Books and Pamphlets Received .......... 539
SUMMARY:
Obstetrics .......... 540
Practical Medicine .......... 544
Surgery .......... 548
Therapeutics .......... 551
MEDICAL NEWS AND ITEMS .......... 555
TAYUYA—A Card to the Profession .......... 559
ANNOUNCEMENTS FOR THE MONTH .......... 560

